# METABOLISM AND AGING

**DOI:** 10.1093/geroni/igad104.0593

**Published:** 2023-12-21

**Authors:** Joseph Baur

## Abstract

Reducing energy intake in the absence of malnutrition (caloric restriction) has long been known as the most robust and reproducible way to experimentally increase lifespan in laboratory models. However, our understanding of which metabolites and nutrient-sensing pathways interact with longevity and where they act remains rudimentary, and in many cases, controversial. This session will explore recent findings linking changes in metabolites to the aging process and to organismal health.

